# Are you bothered? Assessing the association between symptoms and botheredness using real-world data to help improve mental health services

**DOI:** 10.3389/frhs.2026.1753567

**Published:** 2026-02-18

**Authors:** Timothy A. Carey

**Affiliations:** Centre > Health Equity in Regional and Remote Communities, Central Queensland University, Rockhampton, QLD, Australia

**Keywords:** inappropriate healthcare, mental health services, patient-perspective care, psychological distress, symptom questionnaire, mental health disorder, resampling, embedded clinical research

## Abstract

Healthcare can be inappropriate and waste valuable and finite resources when it is not aligned to the needs and goals of patients. In mental health, although the importance of subjective experience and the patient's perspective is generally recognized, many current clinical measures do not reflect this. Mental health services might become more effective and efficient if the data being collected from patients more closely reflected patients' priorities and preferences. To promote embedded clinical research (EmCR) as well as leveraging its benefits, a service innovation was introduced to investigate the interchangeability of symptom ratings and botheredness ratings. A file audit examined routinely collected real-world data including ratings of impairment as well as ratings of botheredness from a clinical psychology outpatient clinic in a public mental health service. The Work and Social Adjustment Scale was modified and data from 61 files were analysed using resampling methods to explore associations between and interchangeability of the ratings. The data indicated that ratings of impairment and botheredness were strongly correlated. Null hypothesis significance testing, however, provided evidence that impairment and botheredness were not, in general, interchangeable. Current approaches to obtaining mental health information from patients might not be aligned with their priorities. These findings could have implications for making mental health services more effective and efficient by improving the extent to which service delivery is meaningful and useful to patients.

## Introduction

Robust mental health is essential for individuals to thrive and communities to flourish. Alarmingly, recent research indicates that the global economic burden of mental disorders has been grossly underestimated with the revised value of the burden estimated at USD 5 trillion (1). Crucially, the burden is getting worse not better placing unrelenting pressure on health systems and services.

### The scourge of inappropriate healthcare

Improving access to high-quality health services necessitates overcoming the widespread phenomenon of inappropriate healthcare including both the overuse of ineffective treatments and the underuse of effective treatments (2). In terms of mental health, overdiagnosis and overmedicalization are common examples of inappropriate care (3). Of relevance to the current study is the suggestion that one of the drivers of inappropriate care is a failure to fully incorporate the needs and preferences of patients in the design and delivery of services (4).

To assist in eliminating inappropriate care and making the provision of appropriate services more widespread, a model of patient-*perspective* care, rather than the standard patient-*centered* model has been suggested (5). For care to be genuinely appropriate it must, ultimately, help people live lives that have meaning and value *to them*. Thus, problems must be defined from the patient's perspective in terms of the experiences, circumstances, conditions, and events that prevent them from achieving their goals (6, 7).

### The importance of perspective

In general, there is widespread acknowledgement of the impact of perspective (8, 9). Various reform initiatives, for example, such as the recovery movement beginning in the 1980s have emphasised the importance of a first-person perspective. The basis of the recovery movement is a position that it is individuals pursuing their preferred futures rather than reducing or eliminating psychiatric symptoms that is fundamental (10). Yet, despite understanding perspective's importance, evidence indicates that more than 30 years of reform movements have not significantly addressed the challenges faced by people experiencing serious psychological distress (10). Perhaps it is a failure to honour and elevate the significance of a first-person perspective that has led to a plea for greater flexibility in mental health services (MHSs) by the people who access these services (8).

Helping people achieve personally valued goals could be considered a fundamental purpose of MHSs. When considering personal goals, the wisdom of Epictetus expressed in his statement that people “are disturbed not by things, but by the views which they take of things” is instructive. Although Epictetus's statement is most explicitly endorsed in services providing cognitive and cognitive behavioral therapies, its ethos can be identified in any MHS that assists people to develop insights and new perspectives into the dilemmas they face.

The notion that one's view of something is crucial to their response to it is reflected in the way that events such as trauma and voice-hearing are understood. It is recognized, for example, that not everyone will be similarly traumatized by the same event (7). Moreover, voice-hearing is not ubiquitously distressing and, in fact, is a positive experience for some people (11). Recognition of how differently something like voice-hearing can be experienced has prompted calls for practitioners to attend more closely to the views of people reporting those experiences (12).

If the priority of a person's perspective, or their “view of things”, is a reasonable position to adopt, this might suggest that MHSs which target subjective experience directly might be effective and efficient. In this sense, particular symptoms or symptom patterns are not regarded as being objectively indicative of disturbed states. Rather, it is the person's view of their apparent symptoms that is decisive (9).

### A consideration of commonly used mental health questionnaires

It appears that, generally, the sentiments of Epictetus and the understanding in the field of the importance of a first-person perspective are not reflected in the mental health measures that are commonly used in MHSs. Specifically, current instruments typically assess the extent to which particular symptoms such as difficulty sleeping or excessive worrying are occurring but they rarely enquire about a person's view of their sleeping or worrying. Some measures assess more global symptom patterns rather than individual symptoms but a focus on the person's perspective is still absent. Table 1 provides the names and abbreviations of 12 mental health instruments that are widely used internationally in MHSs along with an example item from each one. The items contained in the different measures can vary markedly from one-word items, to items about specific sensations, and also to general states or conditions (see Table 1).

**Table 1:** A selection of measures commonly used to assess mental health and wellbeing with an example item from each measure.

Name of measure	Abbreviation	Example item	Reference
1. Beck Anxiety Inventory	BAI	Numbness or tingling.	(13)
2. BBC Subjective Well-Being Scale	BBC-SWB	Do you feel able to grow and develop as a person?	(14)
3. Beck Depression Inventory-II	BDI-II	I criticize myself for all of my faults.	(15)
4. Clinical Outcomes in Routine Evaluation-Outcome Measure	CORE-OM	I have had difficulty getting to sleep or staying asleep.	(16)
5. Depression Anxiety Stress Scales	DASS	I felt that I was rather touchy.	(17)
6. General Anxiety Disorder-7	GAD-7	Worrying too much about different things.	(18)
7. Kessler Psychological Distress Scale	K10	About how often did you feel restless and fidgety?	(19)
8. Mental Health Quality of Life Questionnaire	MHQoL	I think negatively about myself.	(20)
9. Outcome Questionnaire	OQ-45.2	I feel that something bad is going to happen.	(21)
10. Positive and Negative Affect Schedule	PANAS	Upset.	(22)
11. Patient Health Questionnaire	PHQ-9	Poor appetite or overeating.	(23)
12. World Health Organization Quality-of-Life Scale	WHOQOL-BREF	To what extent do you have the opportunity for leisure activities?	WHO, 1998

What is missing almost exclusively from these measures is any enquiry about the person's perspective of the symptoms on which they are reporting. For example: the PANAS (22) doesn't ask if the person is bothered about being upset over the past week; the DASS (17) doesn't ask if the person is bothered about their touchiness over the past week; the BDI-II (15) doesn't ask if the person is bothered about their appetite during the past two weeks; the CORE-OM (16) doesn't ask if the person is bothered about their ability to cope when things go wrong over the last week; the OQ-45.2 (21) doesn't ask if the person is bothered about having sore muscles over the last week; the K10 (19) doesn't ask if the person is bothered about feeling restless or fidgety in the past four weeks; and the MHQoL (20) doesn't ask if the person is bothered by being gloomy about the future today.

Some items seem to approach the matter of botheredness indirectly by asking about states such as satisfaction or happiness. For example, the WHOQOL-BREF (36) has items such as “How satisfied are you with your capacity to work” and the BBC-SWB (14) includes “Are you happy with your looks and appearance”. It is conceivable though, that there could be situations in which a person is dissatisfied with their capacity to work but not especially bothered by their dissatisfaction. Moreover, people could be not particularly happy about their appearance but not be bothered by that.

Other questionnaires appear to address botheredness more directly. Both the GAD-7 (18) and the PHQ-9 (23) ask, “Over the last two weeks, how often have you been bothered by any of the following problems?” Asking *how often* one has been bothered, however, is not the same as asking *how much* botheredness a person is experiencing. The BAI (13), on the other hand, does instruct people to “Indicate how much you have been bothered by that symptom ….  ” on the rating scale, however, while a response of one means “Mildly, but it didn't bother me much”; a response of two means “Moderately—it wasn't pleasant at times”. Response option two, therefore, seems to indicate that the interpretation of “bothered” might not be completely straightforward with the BAI (13) since “wasn't pleasant” will not be synonymous with “bothered” in all contexts and situations.

#### The clinical importance of perspective

If subjective experience and a first-person perspective are to be embraced, it seems important not to make assumptions about even seemingly straightforward topics such as satisfaction and botheredness. This has important clinical implications because it is not improbable that someone could assign an item a high dissatisfaction rating but not be especially bothered about that particular area. Similarly, a person might have been *frequently* bothered by a symptom over the last few weeks, but the *level* of botheration might have only been minimal. Indeed, in a systematic review and qualitative metasynthesis of sources of distress in first-episode psychosis, it was found that the sources of distress people reported were “diverse and multifaceted” (9, p. 118) and, importantly for the current study, these sources of distress “were substantially different from those routinely recognized and targeted in clinical practice” (9).

Of course, it is entirely possible that it has already been demonstrated that symptom level is a reliable and appropriate proxy for level of botheredness. Empirical evidence of this kind, however, does not appear to be readily available in the published literature. For this reason, it was of interest to establish as a proof-of-concept if, in routine service delivery, assessing symptom levels could be regarded as an acceptable substitute for assessing botheredness. If so, this would mean that service delivery decisions based on self-reported symptom scores would be effective in helping people overcome obstacles to achieving the goals that are important to them. If symptom level is not an accurate indicator of the things that are most bothersome to an individual, however, then MHSs might not be as effective as they could be.

#### The research question and the approach to embedded clinical research (EmCR)

Since empirical evidence could not be located, a decision was made to conduct a file audit as EmCR using real-world data. EmCR is an important complement to implementation and translation initiatives by eliminating the gap between research and practice. Moreover, EmCR has the potential to ehance the evidence-*based* practice movement by creating a culture of evidence-*building* practices (24). EmCR organises systems to enable data collected in routine clinical practice to be used to answer important questions related to service efficiency and effectiveness. In this way, EmCR is an example of measurement-based care (MBC; 25) and learning health systems (26).

The research question for this project was “Are self-reported symptom levels interchangeable with self-reported levels of botheredness?” A detailed data analysis plan was developed and, consistent with previous efforts (27), the collected data were analysed in different ways to provide multiple perspectives in answering the question. Further details are provided in the Data Analysis section below.

## Materials and methods

The study was an EmCR file audit using real-world data that are collected and monitored routinely to promote ongoing improvement and high-quality service provision as part of measurement-based care (25). Work by clinician-researchers can contribute to the body of knowledge in a specific field and accelerate the impact of translational and implementation efforts by closing the divide between research and practice settings.

### Research setting

The audit was conducted on the files contained in the public MHS of a remote Australian town. A psychology outpatient clinic was operated by an experienced clinical psychologist clinician-researcher. Due to the availability of the clinician-researcher, between four and eight one-hour appointments each week were available (for more details about the clinic see (28)). Patients were empowered to book appointments with the clinic receptionist on an as-needed basis according to the patient-led appointment scheduling approach (3, 5, 29). Outcome data were routinely collected using standardised questionnaires at the beginning of every appointment.

### The work and social adjustment scale

While there is an abundance of appropriate and relevant measures that could have been used, for this innovation, adapting the Work and Social Adjustment Scale (WSAS; 30) was considered ideal. The WSAS is widely used in health as a brief global measure of functional impairment (31). In a study of the WSAS's psychometric properties involving comparisons with the PHQ-9 (23) and the GAD-7 (18) using data from 4,835 patients, the WSAS was demonstrated to have high internal reliability and sensitivity to treatment effects (32).

One advantage of the WSAS is that it has only five items so is unlikely to burden respondents. To complete the scale, the respondent is asked to rate from 0 (not at all) to 8 (very severely) how impaired they consider themselves to be in each of five areas. No time frame is specified. Another advantage of the WSAS is that it explicitly asks about impairment. Given that the focus of this research was to investigate the association between symptoms and botheredness, it seemed that impairment might be a term that could be considered closely related to botheredness. Furthermore, the WSAS asks about broad areas of impairment such as home management and close relationships.

### Data collection

People attending clinical psychology appointments completed brief measures at every appointment as part of the standard practice of the clinic. For this audit then, data from the entire population of people referred to the service were used.

### Instrument

As mentioned above, the WSAS was used in this study. For the purposes of this project, the WSAS was modified to directly assess how bothered or distressed a person was about each of the five areas of the WSAS. For example, item one of the WSAS is “Because of my problems, my ability to work is impaired. 0 means not at all impaired and 8 means very severely impaired to the point I can't work”. To this item, “How bothered are you about this level of impairment? 0 means not at all bothered and 8 means very, very bothered”. was added. A similarly worded statement was added to each of the five items of the WSAS. Once the WSAS had been administered, therefore, two numbers were provided for each of the five items.

### Data analysis

As indicated earlier, a detailed data analysis plan was developed to guide interrogation of the data and provide different perspectives to answer the research question. All aspects of data management and analysis were conducted using R version 4.4.0 (33).

The data analysis plan first involved summarizing the data and inspecting the ratings' distributions to assess for normality, extreme scores, and missing values. Correlations of the impaired and bothered ratings were calculated using the Spearman rho coefficient given the non-normality of the impaired and bothered ratings' distributions. The statistical significance of the correlation coefficients was assessed using resampling methods (34). Resampling methods were also used to assess the differences between the ratings and the interchangeability of the ratings. Resampling methods were chosen for data analysis because they are powerful, they have an elegant logic, and they are unconstrained by many of the assumptions of parametric tests (35). Moreover, these methods use simulations to test hypotheses and calculate *p* values.

The resampling approach to hypothesis testing for correlation coefficients assesses the assertion that the magnitude of the observed coefficient could have arisen due to chance and would be found commonly in the population from which the data producing the coefficient were obtained. Using this logic, one of the two correlated variables is sampled without replacement to shuffle the order of the values. Another correlation coefficient is then calculated between the newly ordered variable and the existing variable (34, 35). This procedure is conducted a large number of times (1,000 or 10,000 simulations are common) to produce a distribution of correlation coefficients. With this sample of coefficients, it is straightforward to calculate the proportion of coefficients that are as large or larger than the observed coefficient. This proportion is the *p* value that is reported when assessing significance.

Resampling methods are a very useful way of testing differences between scores such as pre and post scores involved in the delivery of a psychotherapy (35). In this situation, the null hypothesis of no difference assumes that the pre and post scores are interchangeable. Under the null hypothesis, if there is no effect of the treatment then a questionnaire score provided after treatment could just as easily have been a score provided before treatment. The logic here is particularly well suited to the current project. The hypothesis being tested is that impaired ratings are a suitable proxy for bothered ratings. According to this logic, the mean difference between the ratings should approach zero and the observed mean difference should be a common result in the population from which the sample was drawn. This particular resampling technique preserves the pairing of the data but randomly changes the sign of the difference. For this study, the difference between the impaired and bothered ratings were calculated to produce five vectors of difference scores. Then, the values 1 and −1 were randomly sampled with replacement according to the number of difference scores available. The assembly of 1 s and −1 s was multiplied with the difference score vectors and new averages were calculated. Again, this procedure was conducted a large number of times enabling a proportion (*p* value) to be calculated according to how many average differences in the distribution were as large or larger than the observed difference. This particular resampling strategy is consistent with a two-tailed approach to hypothesis testing.

Data, study materials (including the data analysis plan), and R code are available upon request from the author.

## Results

The data were collected from 61 files. For the purpose of this EmCR file audit, the demographic profile of the sample was not considered to be particularly relevant. The issue of concern was the conceptual link between self-reported symptoms and self-reported botheredness. Nevertheless, the age range of patients who completed the WSAS was 20 to 67 years old with a mean age of 38 years. Of the 61 patients completing the WSAS, 29 (47.5%) were female. All of the patients referred to the service had received various diagnoses by their treating psychiatrist including: depression; anxiety; social phobia; obsessive compulsive disorder; borderline personality disorder; schizophrenia; and bipolar disorder. Many patients had more than one diagnosis.

### Data inspection and summary

The data were within the expected ranges for all questionnaire items with no extreme scores and no missing values. Differences between the means and medians for the individual questionnaire items indicated that the distributions might not approximate the normal distribution. A histogram for the age of the patients, for example, was positively skewed.

### Bar charts and boxplots

**Figure 1:**
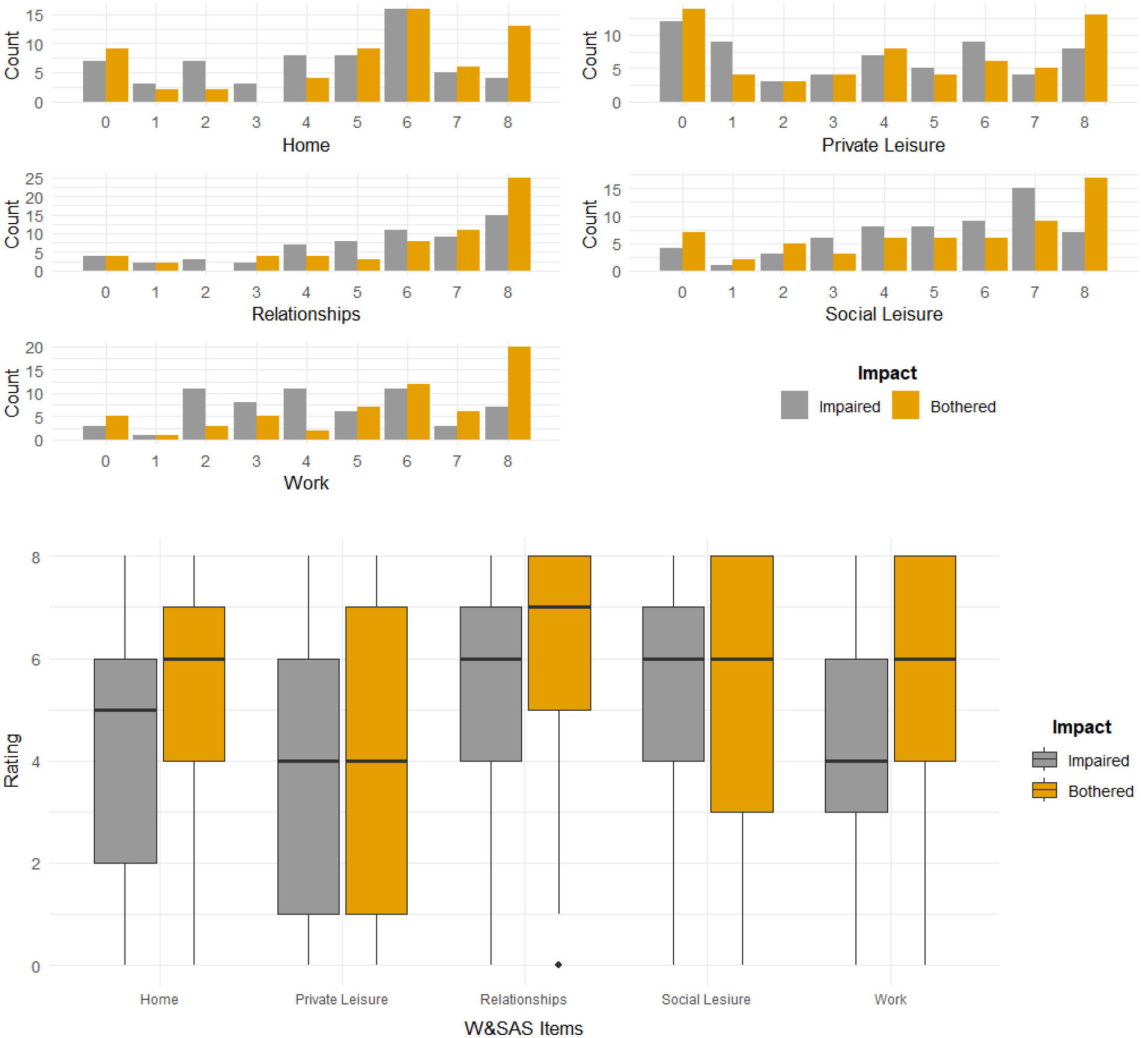
Bar charts and boxplots of impaired and bothered ratings for WSAS items.

The colours used in the bar charts and boxplots were selected from a colour-blind-friendly palette. Bar charts for the individual WSAS items confirmed the non-normality of the distributions. Both impaired and bothered ratings were included in the bar charts to enable comparisons to be made (see Figure 1). Figure 1 indicates that there are some similarities in the ratings as well as substantial heterogeneity.

Boxplots allowed further comparisons to be made between the impaired and bothered ratings while also providing a different perspective of the distribution of the patients' ratings. The medians were different on three (home management, close relationships, work) out of the five items with the bothered median being higher on all occasions (see Figure 1). The range of the central 50% of the ratings was larger for the bothered ratings on three (private leisure, social leisure, work) out of the five items and larger for the impaired rating on the home management item. The boxplots confirm the similarity along with the heterogeneity of the item ratings found with the bar charts.

### Correlation analysis

Correlation coefficients were calculated for the combined set of impaired and bothered ratings as well as for each of the five individual items (see Table 2). Permutation tests were used to assess the significance of the coefficients. In a distribution of 10,000 samples, none of the coefficients were as large or larger than the observed coefficients providing strong evidence for reliable associations between ratings of impairment and ratings of botheredness.

**Table 2:** Spearman Rho correlation coefficients quantifying the association between impaired and bothered ratings for the overall scale as well as individual items*.*

WSAS ratings	Correlation coefficient	Significance
Combined	0.6631	*p* < 0.0001
Home	0.7081	*p* < 0.0001
Private Leisure	0.7558	*p* < 0.0001
Relationships	0.5721	*p* < 0.0001
Social Leisure	0.6379	*p* < 0.0001
Work	0.5596	*p* < 0.0001

### Paired data analysis

The relationship between impaired and bothered ratings were investigated further using resampling methods that preserved the pairing of each of the ratings. Impaired and bothered ratings for each of the items are not independent since each person provided a rating for both impairment and botheredness. As explained above, under the assumption that ratings of impairment are an appropriate substitute for ratings of botheredness, it should be the case that the mean difference between ratings would approach zero and that the mean differences observed in this study would not be uncommon in a much larger distribution of mean difference scores. Table 3 provides the mean difference scores for this study along with the results of null hypothesis significance testing using 10,000 samples. In every case except for the social leisure item, the null hypothesis of no difference was rejected (see Table 3). Therefore, there was strong evidence in this study for the alternative hypothesis that the average mean differences were unusual and that it might not be defensible to assume ratings of impairment are appropriate substitutes for ratings of botheredness.

**Table 3:** Resampling results to test the null hypothesis of the interchangeability of impaired and bothered ratings using 10,000 samples.

WSAS ratings	Mean difference	Significance
Combined	−0.6000	*p* = 0.0001
Home	−0.7869	*p* = 0.0002
Private Leisure	−0.3770	*p* = 0.0013
Relationships	−0.6393	*p* = 0.0097
Social Leisure	0.0328	*p* = 0.9512
Work	−1.2295	*p* = 0.0001

## Discussion

This EmCR file audit project sought to answer the question “Are self-reported symptom levels interchangeable with self-reported levels of botheredness?” The WSAS was modified, and real-world data collected and analysed in different ways to provide a comprehensive answer to the question. Generally, it can be concluded from these results that, even though ratings of impairment and botheredness are strongly correlated, they are not interchangeable. There was some evidence that ratings of social leisure impairment might indicate equivalent levels of botheredness but for all other areas, including the combined WSAS score, there was strong evidence that impaired and bothered ratings were not exchangeable.

### Service delivery implications

These results, potentially, have important implications for MHSs and also provide a useful and relevant example of the importance of MBC (25). If symptom rating levels do not directly reflect the important areas in which an individual is bothered, then service delivery decision-making might be informed by inaccurate information. A reliance on symptom level data could mean that MHSs might be less effective and efficient than they otherwise could be.

MHSs therefore, appear to rely on assuming that self-reported symptom scores accurately reflect a person's perspective about the things they are troubled by. The current results suggest that this might not be an empirically defensible assumption. The value of considering different rating patterns is more evident at an individual level rather than a general, aggregated level. Table 4 provides hypothetical ratings on the home and work items for six respondents. While these data are indicative of the patterns found in this study, the data here do not represent any specific individuals. The ratings clearly demonstrate that sometimes impaired ratings can be high and bothered ratings low, at other times they can be the same, and on other occasions, impaired ratings can be low and bothered ratings can be high (see Table 4). The table provides a stark illustration of the problems that might arise in determining what the clinical implications of impaired ratings might be in the absence of information about botheredness.

**Table 4:** Patterns of impaired and bothered ratings for home and work items.

Home item		Work item	
Impaired	Bothered	Impaired	Bothered
6	8	5	5
8	8	2	8
4	6	4	6
3	6	4	8
6	3	2	5
5	8	5	4

Perhaps a more direct focus on botheredness would enable MHSs to be more targeted and, ultimately, more effective and efficient. Sharpening this focus would be an important step in moving towards working within a framework of patient-perspective care (5) and demonstrating a tangible commitment to a first-person perspective (9, 10). The current results suggest that some of what is experienced in service delivery as ambivalence and resistance could perhaps arise from a misalignment of clinician and patient preferences and priorities.

### Limitations of the study

This project has limitations which somewhat constrain the conclusions that can be drawn. Only one questionnaire was used in one MHS. It may be that different results would be obtained in different settings using different questionnaires. Nevertheless, given that impairment in general areas seems to logically imply insights into botheredness, it might be the case that other questionnaires are even less interchangeable than the WSAS. It is possible that a different pattern of results might emerge from a larger sample. It is also the case that the sample was not from one diagnostic group and perhaps the results might apply differentially to different diagnostic categories. As an initial investigation into the conceptual link between symptoms and botheredness, however, a sample size of 61 is adequate for statements of statistical significance to be made and diagnostic heterogeneity might even be advantageous. For example, it may be that botheredness is an important transdiagnostic construct that has relevance across different diagnoses. While it is important to acknowledge these limitations when considering the conclusions and clinical implications, it might also be helpful to consider that the limitations that have been identified provide useful indicators for future research.

### Future research

Interesting and exciting possibilities for future research have been raised in this project. Different questionnaires could be modified to include botheredness ratings to enable further examination of the possible interchangeability of the ratings. The clinical usefulness of botheredness ratings could also be explored. Are ratings of botheredness more helpful in designing MHSs than ratings of symptoms? Are MHSs more effective and efficient when they are informed by botheredness ratings rather than ratings of symptoms? Do patients report greater satisfaction of MHSs if their botheredness is addressed directly as a priority rather than their apparent symptoms?

## Concluding comments

Adding to the growth of EmCR to improve the effectiveness and efficiency of MHSs as a contribution to reducing the global burden of mental health problems, an investigation into the association between symptoms and degree of botheredness or distress was conducted. While self-reported symptom levels appear to be strongly associated in a correlational sense with self-reported levels of botheredness, the ratings do not appear to be interchangeable. Thus, identifying which symptoms occur most frequently will not necessarily reveal what is most bothersome and in most urgent need of being addressed from the patient's perspective.

Developing treatments that focus more directly on areas of greatest botheredness might help to improve the effectiveness and efficiency of treatment. Recognition of the importance of botheredness could also help to provide new measures that more accurately reflect a person's current subjective experience. By combining the value of EmCR using real-world data along with a genuine appreciation of the importance of patient-perspective care, we might be able to demonstrate greater impact on the global burden of mental health problems for the benefit of individuals and communities.

## Data Availability

The raw data supporting the conclusions of this article will be made available by the author, without undue reservation.
